# Supplementary material for: Structural basis for cytoplasmic dynein-1 regulation by Lis1
Source: eLife. 2022 Jan 7;11:e71229. doi: 10.7554/eLife.71229 (PMC8824474; doi:10.7554/eLife.71229)
Supplement: Supplementary file 1. — A table of the S. cerevisiae strains used in this study. DHA and SNAP refer to the HaloTag (Promega) and SNAP-tag (NEB), respectively. TEV indicates a Tev protease cleavage site. PGAL1 denotes the galactose promoter, which was used for inducing strong expression of Lis1 and dynein motor domain constructs. Amino acid spacers are indicated by g (glycine) and gs (glycine-serine). [file elife-71229-supp1.docx]

**Supplementary file 1. *S. cerevisiae* strains used in this study.**

*DHA* and *SNAP* refer to the HaloTag (Promega) and SNAP-tag (NEB), respectively. TEV indicates a Tev protease cleavage site. *P_GAL1_* denotes the galactose promoter, which was used for inducing strong expression of Lis1 and dynein motor domain constructs. Amino acid spacers are indicated by g (glycine) and gs (glycine-serine).

|  | | |
| --- | --- | --- |
| Strain | Genotype | Source |
| RPY1 | W303a (*MATa; his3-11,15; ura3-1; leu2-3,112; ade2-1; trp1-1*) | (Eshel et al., 1993) |
| RPY799 | W303a; *pep4Δ::HIS5; prb1Δ; dyn1Δ::CgLEU2; GAL1-8HIS-ZZ-SNAPgs-PAC1* | (Huang et al., 2012) |
| RPY816 | W303a; *pep4Δ::HIS5; prb1Δ; GAL1-8HIS-ZZ-Tev-PAC1; dyn1Δ::CgLEU2; ndl1Δ::HPH* | (Huang et al., 2012) |
| RPY1042 | W303a; *pep4Δ::HIS5; prb1Δ; GAL1-8HIS-ZZ-Tev-pac1(aa 3-129 delete)-g-1XFLAG-gaSNAP-kanR; dyn1Δ::CgLEU2; ndl1Δ::HPH* | (Huang et al., 2012) |
| RPY1167 | W303a; *pep4Δ::HIS5; pGAL-ZZ-TEV-GFP-3XHA-GST- DYN1_331kDa_-gsDHA-KanR; prb1Δ; pac1Δ; ndl1Δ::CgLEU2* | (Huang et al., 2012) |
| RPY1302 | W303a; *PGal:ZZ:Tev:DYN1_331kDa_ pep4D::HIS5; prb1Δ PAC11-13Myc-TRP; pac1Δ::HPH* | (Toropova et al., 2014) |
| RPY 1385 | MATa *lys2-801 leu2-Δ1 his3-Δ200 trp1-Δ63 DYN1-3XGFP::TRP1, ura3-52::CFP-TUB1::URA3, SPC110-tdTomato::SpHIS5, ura3Δ::KanMX* | (Toropova et al., 2014) |
| RPY1547 | W303a; *pep4Δ::HIS5; prb1Δ; GAL1-8HIS-ZZ-Tev-pac1(R275A,R301A,R378A,W419A,K437A; dyn1Δ::CgLEU2; ndl1Δ::HPH* | (DeSantis et al., 2017) |
| RPY1717 | *MATa lys2-801; leu2- Δ 1; his3- Δ 200; trp1- Δ 63; DYN1-3XGFP::TRP1; ura3-52::CFP-TUB1::URA3; SPC110-tdTomato::SpHIS5; ura3∆::KanMX; pac1∆::KlURA3* | (DeSantis et al., 2017) |
| RPY1749 | W303a; *pep4Δ::HIS5; prb1Δ; GAL1-8HIS-ZZ-Tev-pac1(S248Q); dyn1Δ::CgLEU2; ndl1Δ::HPH* | This work |
| RPY1751 | W303a; *pep4Δ::HIS5; prb1Δ; GAL1-8HIS-ZZ-Tev-pac1(E253A-H254A); dyn1Δ::CgLEU2; ndl1Δ::HPH* | This work |
| RPY1758 | W303a; *pep4Δ::HIS5; pGAL-ZZ-TEV-GFP-3XHA-GST- dyn1_331kDa_(Δ AA3475-3476)-gsDHA-KanR; prb1Δ; pac1Δ; ndl1Δ::CgLEU2* | This work |
| RPY1760 | W303a; *pep4Δ::HIS5; prb1Δ; GAL1-8HIS-ZZ-Tev-pac1(F185D-I189D-R494A); dyn1Δ::cgLEU2; ndl1Δ::HPH* | This work |
| RPY1790 | *W303a; PGal:ZZ:Tev:D6-dyn1(Δ AA3475-3476); pep4Δ::HIS5; prb1Δ; PAC11-13Myc-TRP; pac1Δ::HPH* | This work |
| RPY1791 | W303a; *pep4Δ::HIS5; prb1Δ; GAL1-8HIS-ZZ-Tev-SNAPgs-pac1(E253A-H254A); dyn1Δ::cgLEU2; ndl1Δ::HPH* | This work |
| RPY1792 | W303a; *pep4Δ::HIS5; prb1Δ; GAL1-8HIS-ZZ-Tev-SNAP-pac1(F185D-I189D-R494A); dyn1Δ::cgLEU2; ndl1Δ::HPH* | This work |
| RPY1793 | W303a; *pep4Δ::HIS5; prb1Δ; GAL1-8HIS-ZZ-Tev-SNAP-pac1(S248Q); dyn1Δ::CgLEU2; ndl1Δ::HPH* | This work |
| RPY1795 | *MATa lys2-801; leu2-Δ1; his3-Δ200; trp1-Δ63; DYN1-3XGFP::TRP1; ura3-52::CFP-TUB1::URA3; SPC110-tdTomato::SpHIS5; ura3∆::KanMX; Flag-PAC1* | This work |
| RPY1796 | *MATa lys2-801; leu2-Δ1; his3-Δ200; trp1-Δ63; DYN1-3XGFP::TRP1; ura3-52::CFP-TUB1::URA3; SPC110-tdTomato::SpHIS5; ura3∆::KanMX; Flag-pac1(E253A-H254A)* | This work |
| RPY1797 | *MATa lys2-801; leu2-Δ1; his3-Δ200; trp1-Δ63; DYN1-3XGFP::TRP1; ura3-52::CFP-TUB1::URA3; SPC110-tdTomato::SpHIS5; ura3∆::KanMX; Flag-pac1(S248Q)* | This work |
| RPY1798 | *MATa lys2-801; leu2-Δ1; his3-Δ200; trp1-Δ63; DYN1-3XGFP::TRP1; ura3-52::CFP-TUB1::URA3; SPC110-tdTomato::SpHIS5; ura3∆::KanMX; Flag-pac1(F185D-I189D-R494A)* | This work |
| RPY 1827 | *MATa lys2-801; leu2-Δ1; his3-Δ200; trp1-Δ63;dyn1(D2868K)- 3XGFP::TRP1; ura3-52::CFP-TUB1::URA3; SPC110-tdTomato::SpHIS5; ura3∆::KanMX* | This work |
| RPY1828 | *MATa lys2-801; leu2-Δ1; his3-Δ200; trp1-Δ63;dyn1(D2868K)- 3XGFP::TRP1; ura3-52::CFP-TUB1::URA3; SPC110-tdTomato::SpHIS5; ura3∆::KanMX; pac1(E253A-H254A)* | This work |
