# Supplementary material for: Structural basis for cytoplasmic dynein-1 regulation by Lis1
Source: eLife. 2022 Jan 7;11:e71229. doi: 10.7554/eLife.71229 (PMC8824474; doi:10.7554/eLife.71229)
Supplement: Supplementary file 2. — Microscopy and model refinement information for dyneinE2488Q– (Lis1)2 dataset and structure. [file elife-71229-supp2.docx]

**Supplementary file 2. Cryo-EM data collection parameters and model refinement statistics.**

|  | Dynein AAA3 Walker B mutant (E2488Q) bound to Lis1 |
| --- | --- |
| **Models**  PDB  EMDB  **Data collection and processing** | 7MGM  23829 |
| Microscope  Camera | Titan Krios  Gatan K2 Summit |
| Voltage (kV) | 300 |
| Electron exposure (e–/Å^2^) | 58.3 |
| Magnification  Defocus range (μm) | 2 – 2.7 |
| Pixel size (Å) | 1.31 |
| **Reconstruction**  Symmetry imposed | C1 |
| Initial particle images (no.) | 561 397 |
| Final particle images (no.) | 83 975 |
| Micrographs collected (no.)  Map resolution (Å) (0.143 FSC threshold) | 2378  3.1 |
|  |  |
| **Model Refinement** |  |
| Initial model used (PDB code) | 5NUG |
| Map-to-model resolution  (0.5 FSC threshold) (Å)  Sphericity (3D FSC) | 3.2  0.972 |
| Map sharpening *B* factor (Å^2^)  Model to Map Fit  CC mask  CC volume  CC peaks | 78.77  0.87  0.87  0.83 |
| *Model composition*  Non-hydrogen atoms  Protein residues  Ligands | 24 080  2964  4 |
| *B* factors (Å^2^)  Protein  Ligand | 74.23  56.39 |
| *R.m.s. deviations*  Bond lengths (Å)  Bond angles (°) | 0.004  0.481 |
| *Validation*  MolProbity score  Clashscore  Poor rotamers (%) | 1.40  5.74  0.00 |
| *Ramachandran*  Favored (%)  Allowed (%)  Disallowed (%) | 97.60  2.40  0.00 |
